# Effect of L-shaped heat source and magnetic field on heat transfer and irreversibilities in nanofluid-filled oblique complex enclosure

**DOI:** 10.1038/s41598-021-95803-z

**Published:** 2021-08-12

**Authors:** Xiao-Hong Zhang, Tareq Saeed, Ebrahem A. Algehyne, M. A. El-Shorbagy, Adel M. El-Refaey, Muhammad Ibrahim

**Affiliations:** 1 College of Science, Hunan City University, Yiyang, 413000 People’s Republic of China; 2 Nonlinear Analysis and Applied Mathematics (NAAM)-Research Group, Department of Mathematics, Faculty of Science, King Abdulaziz University, P.O. Box 80203, Jeddah, 21589 Saudi Arabia; 3 Department of Mathematics, Faculty of Science, University of Tabuk, P.O.Box741, Tabuk, 71491 Saudi Arabia; 4 Department of Mathematics, College of Science and Humanities in Al-Kharj, Prince Sattam Bin Abdulaziz University, Al-Kharj, 11942 Saudi Arabia; 5 Department of Basic Engineering Science, Faculty of Engineering, Menoufia University, Shebin El-Kom, 32511 Egypt; 6 Department of Basic and Applied Science, College of Engineering and Technology, Arab Academy for Science, Technology & Maritime Transport, Smart Village Campus, Cairo, Egypt; 7 School of Mathematics and Physics, University of Science and Technology Beijing, Beijing, 100083 People’s Republic of China; 8 Nanotechnology Research Unit (NRU), University of Tabuk, Tabuk, 71491 Saudi Arabia

**Keywords:** Energy science and technology, Engineering, Nanoscience and technology

## Abstract

In this paper, the natural convection heat transfer of water/alumina nanofluid is investigated in a closed square cavity. An oblique magnetic field is applied on the cavity of angle $\gamma$. There is also radiation heat transfer in the cavity. The cavity includes a high-temperature source of L-shape. A low-temperature source as a quadrant of a circle is placed at the corner of the cavity. All other walls are well insulated. The novelty of this work is a low-temperature obstacle embedded in the cavity. Simulations are conducted with a Fortran code based on the control volume method and simple algorithm. Entropy generation rate, Bejan number, and heat transfer are studied by changing different parameters. Results indicate that the highest rates of heat transfer and entropy generation have occurred for the perpendicular magnetic field at high values of the Rayleigh number. At these Rayleigh numbers, the minimum value of the Bejan number is obtained for 15° magnetic field. The magnetic field variation can lead to a change up to 53% in Nusselt number and up to 34% in generated entropy. In a weak magnetic field, the involvement of the radiation heat transfer mechanism leads to an increase in the heat transfer rate so that the Nusselt number can be increased by ten units considering the radiation heat transfer when there is no magnetic field. The maximum heat transfer rate occurs in the horizontal cavity and the minimum value in the cavity of 60° angle. For water, these values are 10.75 and 9.98 for 0 and 60 angles, respectively. Moreover, a weak magnetic field increases the heat transfer rate in the absence of the radiation mechanism, while it is reduced by considering a strong magnetic field.

## Introduction

In recent years, researchers have continuously sought ways to enhance thermal performance in various thermal types of equipment. These are heat exchangers, heat sinks, channels, and cavities. Among the mentioned equipment, cavities have drawn the attention of researchers due to their plentiful applications in diverse industries. Researchers have utilized various methods to raise the heat transfer rate. Using nanofluid and attaching fins are two common ways of improving the heat transfer rate. The addition fins can result in an increase in heat transfer by enlarging the surface through which heat is exchanged^1–5^. Because of their higher thermal conductivity, adding nanopowders can increase the heat transfer rate of the production. According to previous studies, using nanofluids can increase the heat transfer rate^6–13^. As a result, many researchers have studied the simultaneous presence of a nanofluid and fins in a cavity. Alrashed et al.^14^ have investigated a cavity with two fins attached. By studying the effect of different fin angles on the thermal performance, they have discovered that using nanofluid has a positive effect, thermally. Pordanjani et al.^15^ have researched the influence of the simultaneous presence of nanofluid and two fins in a square cavity. They have found out that adding more nanopowder improves the thermal performance.

In industry, the existence of electric currents can lead to the creation of magnetic fields in the proximity of cavities. Numerous researchers have studied the effect of magnetic fields, which may affect the flow field because of the electric conductivity of nanoparticles^16–23^. Dogonchi et al.^24^ have investigated the free heat convection inside the typical copper–water nanofluid filled-enclosure via the CVEFM method.

Radiative heat transfer is another heat transfer mechanism and has gained less attention than convective heat transfer from researchers. This type of heat transfer has found widespread application in numerous industries related to solar energy or those involving high temperatures. Further research is therefore required on this type of heat transfer. Some researchers have investigated radiative heat transfer in cavities in the past^25–30^. Yan et al.^31^ included radiation phenomenon in an alumina-water nanofluid-filled cavity. In this research, a rectangular fin was attached to the left wall. They studied Ra and Ha numbers, the nanofluid concentration, and the radiation parameter. They found out that the addition of radiation to the cavity raises the thermal performance and irreversibilities. Some researchers used numerical methods for their studies^32–36^

The energy consumed by humankind is increasing every year, resulting in harmful environmental impacts. In addition, the burning of fossil fuels is on the rise, and researchers have been seeking solutions for reducing the energy consumption of pieces of equipment, given the limitation of nonrenewable energy sources. For the purpose of improving efficiency, one method is measuring the entropy generation and irreversibilities which was studied by researchers^37–42^. Afrand et al.^43^ did so in a square cavity. They discovered that rising/declining the Ra/Ha numbers leads to a rise of irreversibilities and a fall in the Bejan number.

Given the rise in global energy consumption, researchers have tried to enhance heat transfer in thermal equipment such as closed cavities. Two solutions, namely the use of nanofluids and fins, have been suggested by many researchers in recent years for improving the thermal performance. However, attaching fins investigated in the majority of researches, especially those involving coding, have had simple geometrical shapes. This is while, in practice, various geometrical shapes are used in the industry which needs to be studied. One of these shapes is the circular shape which is widely used in the industry. Pipes are an instance of equipment with a circular cross-section and are indispensable in industry. The presence of electric currents and therefore magnetic fields in various industries has led to a concern to investigate the influence of magnetic fields on different equipment. One form of energy extractable by mankind is solar energy which can be a suitable substitute for fossil fuels. Since heat transfer in equipment involved with solar energy is in the form of radiation, further research on this type of clean heat transfer is imperative. Finally, it is advisable that the efficiency of heat transfer equipment such as cavity be examined in order to endeavor to improve this efficiency. Given the mentioned issues, MHD free heat convection and entropy generation accompanied by radiation in an alumina-water nanofluid-filled enclosure is investigated in this paper. A quarter-circular arc-shaped fin is located at the top corner of the right wall. Unlike the fins in most papers that are at high temperatures, the fin in this work is at a low temperature. The variable parameters in this paper are the Ha and Ra numbers, cavity angle, magnetic field angle, nanofluid concentration, and radiation parameter.

## Problem statement

**Figure 1:**
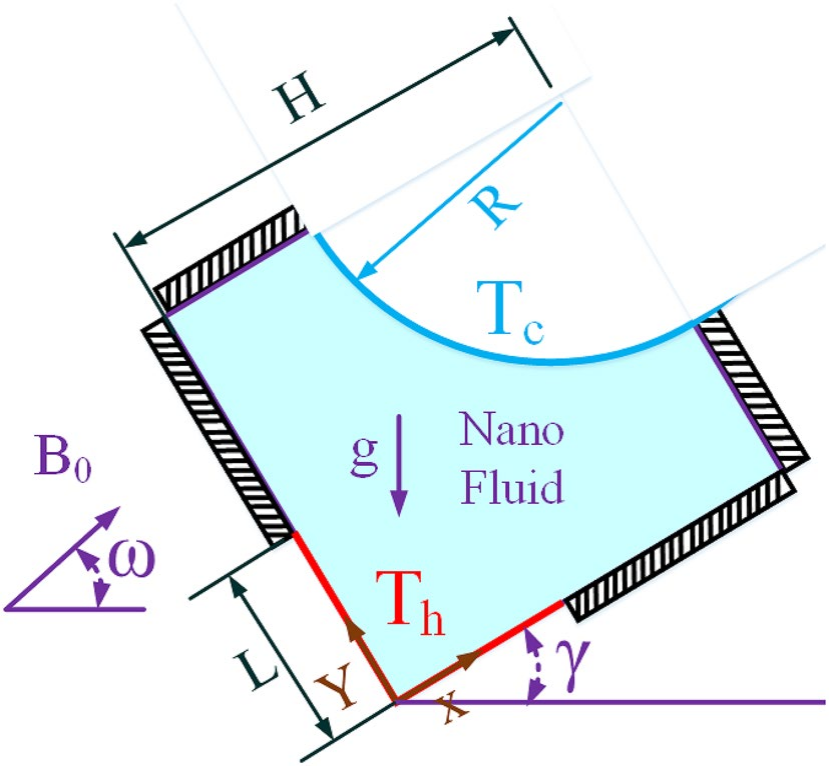
Geometry of the problem.

As shown in Fig. 1, the problem schematic consists of a square enclosure with the length of H. The Al_2_O_3_-water nanofluid-filled enclosure is positioned at an angle of γ. A magnetic field with an intensity of B_0_ and an angle of ω with the horizon is incident on the cavity. A fin in the shape of a quarter-circular arc and at a temperature of T_c_ is located at the top-right. A section with the length of L from the lower part of the left wall is at a temperature of T_h_ and other walls are insulated.

### Governing equations

The equations are derived for a steady, laminar, incompressible, and Newtonian nanofluid flow. Viscous dissipation has been ignored and density variation has been modeled using Boussinesq approximation. The first group of equations written in small letters is dimensional, and the second group of equations written in capital letters and Italic script is dimensionless^43,44^.

Equation of conservation of mass

1 $$\frac{\partial \mathrm{u}}{\partial \mathrm{x}}+\frac{\partial \mathrm{v}}{\partial \mathrm{y}}=0$$

2 $$\frac{\partial U}{\partial X}+\frac{\partial V}{\partial Y}=0$$

Equation of conservation of momentum along the x-direction

3 $$\mathrm{u}\frac{\partial \mathrm{u}}{\partial \mathrm{x}}+\mathrm{v}\frac{\partial \mathrm{u}}{\partial \mathrm{y}}=-\frac{1}{\uprho }\frac{\partial \mathrm{p}}{\partial \mathrm{x}}+\frac{1}{{\uprho }_{\mathrm{nf}}}\left(\frac{\partial }{\partial \mathrm{x}}({\upmu }_{\mathrm{nf}}\frac{\partial \mathrm{u}}{\partial \mathrm{x}})+\frac{\partial }{\partial \mathrm{y}}({\upmu }_{\mathrm{nf}}\frac{\partial \mathrm{u}}{\partial \mathrm{y}})\right)+\frac{\upsigma {\mathrm{B}}^{2}}{\uprho }(\mathrm{vsin\omega }.\mathrm{cos\omega }-{\mathrm{usin}}^{2}\upomega )+\mathrm{g}{\upbeta }_{\mathrm{nf}}(\mathrm{T}-{\mathrm{T}}_{\mathrm{c}})\mathrm{sin\gamma }$$

4 $$U\frac{\partial U}{\partial X}+V\frac{\partial U}{\partial Y}=-\frac{\partial P}{\partial X}+\frac{{\mu }_{nf}}{{\rho }_{nf}{\alpha }_{f}}\left(\frac{{\partial }^{2}U}{{\partial X}^{2}}+\frac{{\partial }^{2}U}{{\partial Y}^{2}}\right)+\frac{{\rho }_{f}}{{\rho }_{nf}}\frac{{\sigma }_{nf}}{{\sigma }_{f}}PrH{a}^{2}(Vsin\omega cos\omega -{Usin}^{2}\omega )+\frac{{\beta }_{nf}}{{\beta }_{f}}RaPr\theta sin\gamma$$

Equation of conservation of momentum along the y-direction

5 $$\mathrm{u}\frac{\partial \mathrm{v}}{\partial \mathrm{x}}+\mathrm{v}\frac{\partial \mathrm{v}}{\partial \mathrm{y}}=-\frac{1}{{\uprho }_{\mathrm{nf}}}\frac{\partial \overline{\mathrm{P}}}{\partial \mathrm{y} }+\frac{1}{{\uprho }_{\mathrm{nf}}}\left(\frac{\partial }{\partial \mathrm{x}}\left({\upmu }_{\mathrm{nf}}\frac{\partial \mathrm{v}}{\partial \mathrm{x}}\right)+\frac{\partial }{\partial \mathrm{y}}\left({\upmu }_{\mathrm{nf}}\frac{\partial \mathrm{v}}{\partial \mathrm{y}}\right)\right)+\frac{{\upsigma }_{\mathrm{nf}}{\mathrm{B}}^{2}}{{\uprho }_{\mathrm{nf}}}(\mathrm{usin\omega cos\omega }-{\mathrm{vcos}}^{2}\upomega )+\mathrm{g}{\upbeta }_{\mathrm{nf}}(\mathrm{T}-{\mathrm{T}}_{\mathrm{c}})\mathrm{cos\gamma }$$

6 $$U\frac{\partial V}{\partial X}+V\frac{\partial V}{\partial Y}=-\frac{\partial P}{\partial Y}+\frac{{\mu }_{nf}}{{\rho }_{nf}{\alpha }_{f}}\left(\frac{{\partial }^{2}V}{{\partial X}^{2}}+\frac{{\partial }^{2}V}{{\partial Y}^{2}}\right)+\frac{{\rho }_{f}}{{\rho }_{nf}}\frac{{\sigma }_{nf}}{{\sigma }_{f}}PrH{a}^{2}\left(Usin\omega cos\omega -{Vcos}^{2}\omega \right)+\frac{{\beta }_{nf}}{{\beta }_{f}}RaPr\theta cos\gamma$$

Equation of conservation of energy

7 $$\left(\mathrm{u}\frac{\partial \mathrm{T}}{\partial \mathrm{x}}+\mathrm{v}\frac{\partial \mathrm{T}}{\partial \mathrm{y}} \right)=\frac{{\mathrm{k}}_{\mathrm{nf}}}{{\left(\uprho {\mathrm{C}}_{\mathrm{P}}\right)}_{\mathrm{nf}}}\left(\frac{{\partial }^{2}\mathrm{T}}{{\partial \mathrm{x}}^{2}}+\frac{{\partial }^{2}\mathrm{T}}{{\partial \mathrm{y}}^{2}}\right)-\frac{1}{\uprho {\mathrm{C}}_{\mathrm{P}}}\frac{\partial {\mathrm{q}}_{\mathrm{r}}}{\partial \mathrm{y}} , \left[{\mathrm{q}}_{\mathrm{r}}=-\frac{4}{3}\frac{{\upsigma }_{\mathrm{e}}}{{\upbeta }_{\mathrm{R}}}\frac{\partial {\mathrm{T}}^{4}}{\partial \mathrm{y}},{\mathrm{T}}^{4}=4{\mathrm{T}}_{\mathrm{c}}^{3}\mathrm{T}-3{\mathrm{T}}_{\mathrm{c}}^{4}\right]$$

8 $$\left(U\frac{\partial \theta }{\partial X}+V\frac{\partial \theta }{\partial Y} \right)=\frac{{\alpha }_{nf}}{{\alpha }_{f}}\left(\frac{{\partial }^{2}\theta }{{\partial X}^{2}}+\frac{{\partial }^{2}\theta }{{\partial Y}^{2}}\right)+\frac{4}{3}\frac{{k}_{nf}/{k}_{f}}{{\left(\rho {C}_{P}\right)}_{nf}/{\left(\rho {C}_{P}\right)}_{f}}{\left(\frac{{k}_{nf}}{{k}_{f}}\right)}^{-1}Rd\frac{{\partial }^{2}\theta }{\partial {Y}^{2}}$$

Equation of entropy generation^43,44^

9 $${\mathrm{S}}_{\mathrm{gen}}=\frac{{\mathrm{k}}_{\mathrm{nf}}}{{\mathrm{T}}_{0}^{2}}\left({\left(\frac{\partial \mathrm{T}}{\partial \mathrm{x}}\right)}^{2}+{\left(\frac{\partial \mathrm{T}}{\partial \mathrm{y}}\right)}^{2}\right)+\frac{{\upmu }_{\mathrm{nf}}}{{\mathrm{T}}_{0}}\left\{2\left[{\left(\frac{\partial \mathrm{u}}{\partial \mathrm{x}}\right)}^{2}+{\left(\frac{\partial \mathrm{v}}{\partial \mathrm{y}}\right)}^{2}\right]+{\left(\frac{\partial \mathrm{u}}{\partial \mathrm{y}}+\frac{\partial \mathrm{v}}{\partial \mathrm{x}}\right)}^{2}\right\}+\frac{{\upsigma }_{\mathrm{nf}}{\mathrm{B}}^{2}}{{\mathrm{T}}_{0}}{\left(\mathrm{usin\omega }-\mathrm{vcos\omega }\right)}^{2}$$

10 $$S_{gen} = \frac{{k_{nf} }}{{k_{f} }}\left( {\left( {\frac{\partial \theta }{{\partial X}}} \right)^{2} + \left( {\frac{\partial \theta }{{\partial Y}}} \right)^{2} } \right) + \zeta \left\{ {2\left[ {\left( {\frac{\partial U}{{\partial X}}} \right)^{2} + \left( {\frac{\partial V}{{\partial Y}}} \right)^{2} } \right] + \left( {\frac{\partial U}{{\partial Y}} + \frac{\partial V}{{\partial X}}} \right)^{2} } \right\} + \zeta \frac{{\sigma_{nf} }}{{\sigma_{f} }}\frac{{\mu_{f} }}{{\mu_{nf} }}Ha^{2} \left( {Usin\omega - Vcos\omega } \right)^{2}$$

The equations governing the nanofluid flow expressed above have been rendered dimensionless via the parameters expressed in Eqs. (11) and (12).

11 $$\mathrm{X}=\frac{\mathrm{x}}{\mathrm{l}}, \mathrm{Y}=\frac{\mathrm{y}}{\mathrm{l}}, \mathrm{U}=\frac{\mathrm{ul}}{{\mathrm{\alpha }}_{\mathrm{f}}}, \mathrm{V}=\frac{\mathrm{vl}}{{\mathrm{\alpha }}_{\mathrm{f}}} , \mathrm{P}=\frac{\overline{\mathrm{P}}{\mathrm{l} }^{2}}{{\uprho }_{\mathrm{nf}}{\mathrm{\alpha }}_{\mathrm{f}}^{2}} ,\uptheta =\frac{\mathrm{T}-{\mathrm{T}}_{\mathrm{c}}}{{\mathrm{T}}_{\mathrm{h}}-{\mathrm{T}}_{\mathrm{c}}} , \mathrm{L}=\frac{l}{\mathrm{l}},\mathrm{ H}=\frac{L}{\mathrm{l}},\mathrm{ R}=\frac{\mathrm{r}}{\mathrm{l}}$$

12 $${\text{Pr}} = \frac{{\vartheta_{{\text{f}}} }}{{\alpha_{{\text{f}}} }},\;{\text{ Ra}} = \frac{{{\text{g}}\beta_{{\text{f}}} {\text{l}}^{3} ({\text{T}}_{{\text{h}}} - {\text{T}}_{{\text{c}}} )}}{{\alpha_{{\text{f}}} \vartheta_{{\text{f}}} }},\;{\text{Ha}} = {\text{B}}_{0} {\text{l}}\sqrt {\frac{{\sigma_{{\text{f}}} }}{{\rho_{{\text{f}}} \vartheta_{{\text{f}}} }}} ,\; \zeta = \frac{{\mu_{{{\text{nf}}}} {\text{T}}_{0} }}{{{\text{k}}_{{\text{f}}} }}\left( {\frac{{\alpha_{{\text{f}}} }}{{{\text{L}}\left( {{\text{T}}_{{\text{h}}} - {\text{T}}_{{\text{c}}} } \right)}}} \right)^{2} ,\;{\text{Bej}} = \frac{{{\text{S}}_{{{\text{gen}},{\text{T}}}} }}{{{\text{S}}_{{{\text{Total}}}} }},\;{\text{Rd}} = \frac{{4{\text{T}}_{{\text{C}}}^{3} }}{{{\text{k}}_{{\text{f}}} }}\frac{{\sigma_{{\text{e}}} }}{{\beta_{{\text{R}}} }}$$

The Nusselt number is used as a criterion for evaluating the heat transfer through the walls.

The local Nusselt number is defined below.

13 $${\mathrm{Nu}}_{\mathrm{Y}}=\frac{\mathrm{hL}}{{\mathrm{k}}_{\mathrm{f}}}+{\mathrm{Nu}}_{\mathrm{Rd}}$$

The convective heat transfer coefficient expressed in the above relationship is defined below.

14 $$\mathrm{h}=\frac{{\mathrm{q}}_{\upomega }}{{\mathrm{T}}_{\mathrm{h}}-{\mathrm{T}}_{\mathrm{c}}}$$

Also, the heat flux is computed from Eq. (15).

15 $${\mathrm{q}}_{\upomega }={\mathrm{k}}_{\mathrm{nf}}\left(\frac{\partial \mathrm{T}}{\partial \mathrm{X}}\right)$$

After simplification, the local and averaged values of Nusselt number are calculated as in Eqs. (16) and (17).

16 $$\begin{gathered} Nu_{x = 0} = - \frac{{k_{nf} }}{{k_{f} }}\left( {\frac{\partial \theta }{{\partial X}}} \right) + \frac{4}{3}Rd\left( {\frac{\partial \theta }{{\partial X}}} \right) = \frac{{k_{nf} }}{{k_{f} }}\left( {1 + \frac{4}{3}Rd\frac{{k_{f} }}{{k_{nf} }}} \right)\frac{\partial \theta }{{\partial X}} \hfill \\ Nu_{y = 0} = - \frac{{k_{nf} }}{{k_{f} }}\left( {\frac{\partial \theta }{{\partial y}}} \right) + \frac{4}{3}Rd\left( {\frac{\partial \theta }{{\partial y}}} \right) = \frac{{k_{nf} }}{{k_{f} }}\left( {1 + \frac{4}{3}Rd\frac{{k_{f} }}{{k_{nf} }}} \right)\frac{\partial \theta }{{\partial y}} \hfill \\ \end{gathered}$$

17 $${Nu}_{M}=\frac{1}{L/2}{\int }_{0}^{L/2}{(Nu}_{(x=0 )}dY+{Nu}_{(y=0 )}dX)$$

Integrating ${\mathrm{S}}_{\mathrm{gen}}$ and the $Be$ number over the whole solution domain.

18 $${S}_{tot}=\int {\mathrm{S}}_{\mathrm{gen}}d\Omega ={\iint }_{0}^{L}{\mathrm{S}}_{\mathrm{gen}}dXdY$$

19 $$Be={\iint }_{0}^{L}BedXdY$$

### Relationships representing the nanofluid properties

The nanofluid properties are determined as follows^43^.

20 $${\upsigma }_{\mathrm{nf}}=\left(1-{\varphi }\right){\upsigma }_{\mathrm{f}}+{\varphi }{\upsigma }_{\mathrm{s}}$$

21 $${\uprho }_{\mathrm{nf}}=\left(1-{\varphi }\right){\uprho }_{\mathrm{f}}+{\varphi }{\uprho }_{\mathrm{s}}$$

22 $$({\mathrm{\rho \beta })}_{\mathrm{nf}}=\left(1-{\varphi }\right)({\mathrm{\rho \beta })}_{\mathrm{f}}+{\varphi }({\mathrm{\rho \beta })}_{s}$$

23 $${(\uprho {\mathrm{c}}_{\mathrm{p}})}_{\mathrm{nf}}=\left(1-{\varphi }\right){(\uprho {\mathrm{c}}_{\mathrm{p}})}_{\mathrm{f}}+{\varphi }{(\uprho {\mathrm{c}}_{\mathrm{p}})}_{\mathrm{s}}$$

24 $${\mathrm{\alpha }}_{\mathrm{nf}}=\frac{{\mathrm{k}}_{\mathrm{nf}}}{{(\uprho {\mathrm{c}}_{\mathrm{p}})}_{\mathrm{nf}}}$$

Considering the Brownian motion, the model proposed by Vajjha^45^ was used. The Maxwell model^46^ was also used for the calculation of ${k}_{Static}$ in the thermal conductivity coefficient.

25 $${\mathrm{k}}_{\mathrm{nf}}={\mathrm{k}}_{\mathrm{Static}}+{\mathrm{k}}_{\mathrm{Brownian}}=\frac{{\mathrm{k}}_{\mathrm{s}}+2{\mathrm{k}}_{\mathrm{f}}-2({\mathrm{k}}_{\mathrm{f}}-{\mathrm{k}}_{\mathrm{s}})}{{\mathrm{k}}_{\mathrm{s}}+2{\mathrm{k}}_{\mathrm{f}}+({\mathrm{k}}_{\mathrm{f}}-{\mathrm{k}}_{\mathrm{s}}){\varphi }}{\mathrm{k}}_{\mathrm{f}}+5\times {10}^{4}\mathrm{\beta \varphi }{\uprho }_{\mathrm{f}}{\left({\mathrm{C}}_{\mathrm{p}}\right)}_{\mathrm{f}}\sqrt{\frac{\mathrm{kT}}{{\uprho }_{\mathrm{s}}{\mathrm{d}}_{\mathrm{s}}}}\mathrm{f}\left(\mathrm{T}, \varphi \right)$$

Considering Brinkman equation^47^ for the static term, the viscosity relationship is expressed as follows.

26 $${\upmu }_{\mathrm{nf}}={\upmu }_{\mathrm{Static}}+{\upmu }_{\mathrm{Brownian}}=\frac{{\upmu }_{\mathrm{f}}}{{\left(1-{\varphi }\right)}^{2.5}}+5\times {10}^{4}{\beta \varphi }{\uprho }_{\mathrm{f}}{\left({\mathrm{C}}_{\mathrm{p}}\right)}_{\mathrm{f}}\frac{{\upmu }_{\mathrm{f}}}{{\mathrm{k}}_{\mathrm{f}}\mathrm{Pr}}\sqrt{\frac{{\mathrm{k}}_{\mathrm{b}}\mathrm{T}}{{\uprho }_{\mathrm{p}}{\mathrm{d}}_{\mathrm{s}}}}\mathrm{f}\left({\mathrm{T }}, \varphi \right)$$

27 $${\text{f}}\left( {{\text{T,}}\varphi } \right) = \left( {2.8217 \times 10^{{ - 2}} \varphi + 3.917 \times 10^{{ - 3}} } \right)\left( {\frac{{\text{T}}}{{{\text{T}}_{0} }}} \right) + \left( { - 3.0669 \times 10^{{ - 2}} \varphi - 3.91123 \times 10^{{ - 3}} } \right)$$

28 $$\upbeta =8.4407{(100{\varphi })}^{-1.07304}$$

The properties of the nanoparticles and the base fluid are given in Table 1.

**Table 1:** Thermophysical properties of ${\mathrm{Al}}_{2}{O}_{3}$-water^45^.

	${C}_{P }(J/kg K)$	$k (W/m K)$	$\uprho (kg/{m}^{3})$	$\upmu (kg/m s)$	$\sigma {\left(\Omega m\right)}^{-1}$	${d}_{S }(nm)$
Water	4179	0.613	997.1	0.001	0.05	–
${Al}_{2}{O}_{3}$	765	40	3970	–	10^–12^	47

### Thermal and hydrodynamic boundary conditions

**Figure 2:**
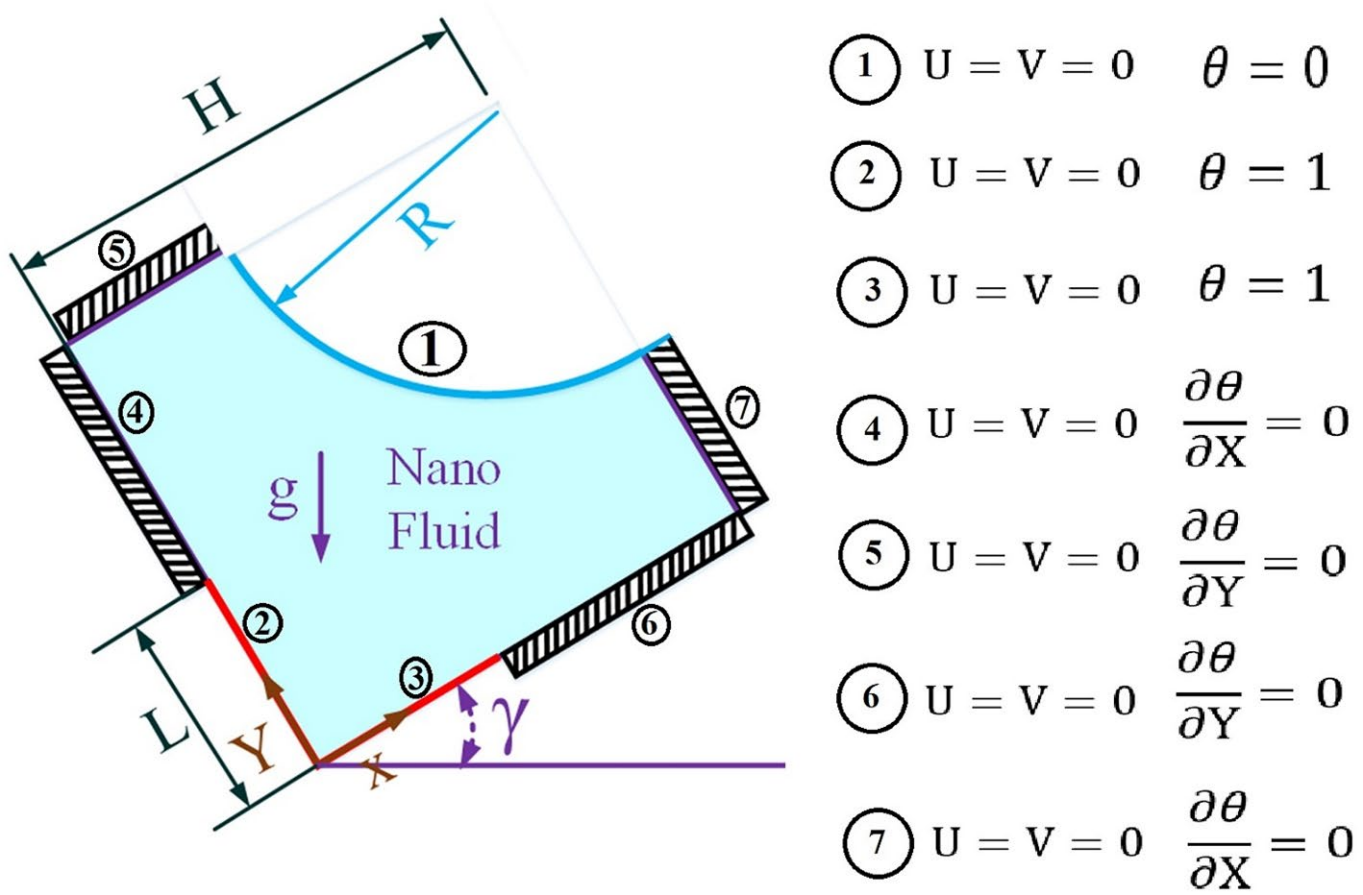
Dimensionless boundary conditions.

The thermal and hydrodynamic wall boundary conditions must be known in order to solve the equations governing the nanofluid flow. These boundary conditions are given in the dimensionless form in Fig. 2.

### Numerical method

The governing equations were discretized using the finite volume method over staggered grid in which the flow characteristics are calculated at the interfaces and the pressure is determined in the middle of grids. The SIMPLE algorithm was used and the solution is stopped if the following convergence criterion is met^48^.

29 $$\Phi =\sum_{\mathrm{J}}\sum_{\mathrm{I}}\left|\frac{{{\varphi }}^{\mathrm{n}+1}-{{\varphi }}^{\mathrm{n}}}{{{\varphi }}^{\mathrm{n}+1}}\right|\le {10}^{-8}$$

## Validation

To evaluate the accuracy of the FORTRAN code, a comparison for the Nu_ave_ was made with the work carried out in Refs^49–51^. These papers deal with the free air convection in the typical square enclosure (Table 2). As seen, the results of the current study differ little from those of the mentioned references.

**Table 2:** Comparison of the Nusselt number in two-dimensional free convection resulting from the computer program and those of other works.

Ra	Ref.^49^	Ref.^51^	Ref.^50^	Present work
10^3^	1.11	1.141	1.085	1.121
10^4^	2.243	2.29	2.100	1.245
10^5^	4.519	4.964	4.361	4.519
10^6^	8.799	–	–	8.808
Max error (%)	0.2	8.9	6.9	0

Moreover, Table 3 shows a comparison between the total entropy results of this paper and those of Oliveski^52^ for the entropy generation inside the typical enclosure for different Ra numbers. The maximum error of 1.65% is observed.

**Table 3:** Comparison of average entropy for different Ra numbers.

$Ra$	${10}^{3}$		${10}^{4}$		${10}^{5}$	
	${S}_{\mathrm{tot}}$	Error	${S}_{\mathrm{tot}}$	Error	${S}_{\mathrm{tot}}$	Error
Oliveski^52^	1.418	1.14%	4.15	0.29%	8.673	1.65%
Present work	1.402		4.138		8.532	

## Meshing study

Nu_ave_ and S_total_ are compared in different grid resolutions to find the appropriate one in which the results become independent of the grid size. According to the results, which were obtained in $\phi =0.03$, $Ra={10}^{5}$, $Ha=20$ and shown in Table 4, the gird sizes larger than 140 × 140 provide no more accuracy.

**Table 4:** The averaged Nusselt number along the cold surface, the maximum stream function and the total entropy for $Ha=20, \, \phi =0.03, \, Ra={10}^{5}$.

Grid	$Ra={10}^{3}$					
	80 × 80	100 × 100	120 × 120	140 × 140	160 × 160	180 × 180
${Nu}_{\mathrm{m}}$	10.822	10.924	10.943	10.950	10.950	10.950
${\Psi }_{max}$	9.548	9.753	9.801	9.818	9.818	9.817
${\mathrm{S}}_{tot}$	5.002	5.071	5.112	5.113	5.113	5.113

## Results and discussion

### Variations in the magnetic field angle and Rayleigh number

**Figure 3:**
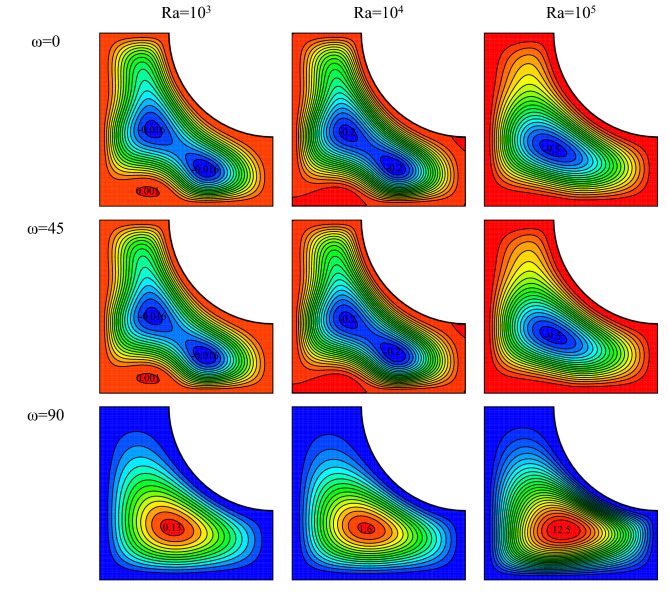
Flow field for $Ha=20, \, Rd=1, \, \varphi =0.03, \, \gamma ={45}^{\circ}$ and various Ra numbers and magnetic field angles.

Figure 3 displays the streamlines for $Ha=20, \, Rd=1, \, \varphi =0.03, \, \gamma ={45}^{\circ}$ and various Ra numbers and magnetic field angles. It is observed that the maximum stream function has raised with rising Rayleigh number. A rise in Rayleigh number is equivalent to a rise in the buoyancy force, which is produced because of the density variation originated from temperature difference. The higher the Rayleigh number is, the larger the temperature difference exerted on the wall becomes, leading to a larger buoyancy force. With a rise in the buoyancy force, the fluid moves faster inside the cavity. This results in a larger vortex velocity and higher stream function values. The change in the magnetic field angle has different effects on the flow field one major of which is on the rotation angle of the vortex in the cavity. The direction of the gravitational acceleration, the magnetic field, and the conditions of vortex formation are important factors that influence the direction and size of a vortex. The rotation direction of the vortex is observed to be clockwise for 0° and 45° magnetic field angles and counterclockwise for a 90° magnetic field angle.

**Figure 4:**
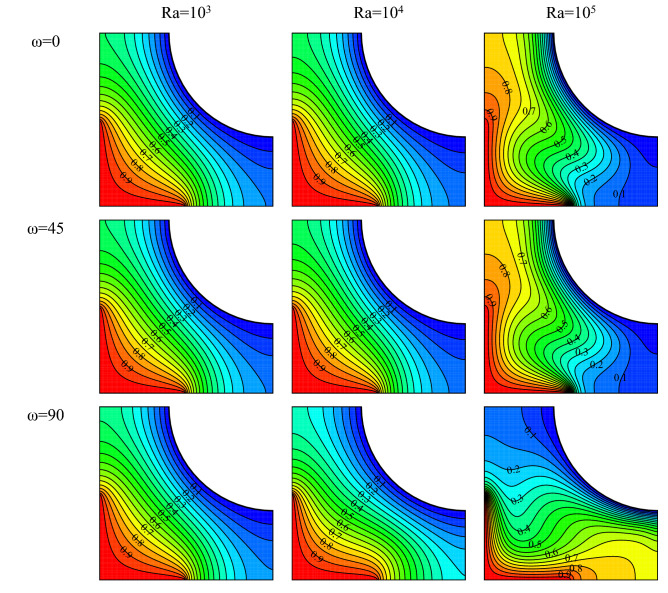
Temperature field for $Ha=20, \, Rd=1, \, \varphi =0.03, \, \gamma ={45}^{\circ}$ and various Rayleigh numbers and magnetic field angles.

Isotherms for different Rayleigh numbers and various ω are drawn in Fig. 4 in $Ha=20, \, Rd=1, \, \varphi =0.03, \, \gamma ={45}^{\circ}$. As shown, isothermal lines become cluttered with a rise in Ra number for all ω. Although these lines are orderly and parallel at low Rayleigh numbers, they increase in curvature and become disorderly at high Rayleigh numbers. This can be explained via the flow field. Vortices rotate faster at higher Rayleigh numbers, results in a rise in free heat convection. At lower Ra numbers, the fluid is almost stationary in the cavity, and the conduction mode becomes dominant. This is clearly observed from the isothermal lines. These lines become cluttered at high Ra numbers, signaling the displacement of the fluid. Variation of ω at low Rayleigh numbers has not considerably affected the isothermal lines. However, it is seen at large Rayleigh numbers that the change in the ω has affected the curvature and the density of lines. Isotherms are almost flat at ω = 45° compared to other values of ω. This is owing to the impact of ω on the intensity and direction of vortices leading to a change in the heat transfer mechanism by strengthening or weakening each mode of heat transfer.

**Figure 5:**
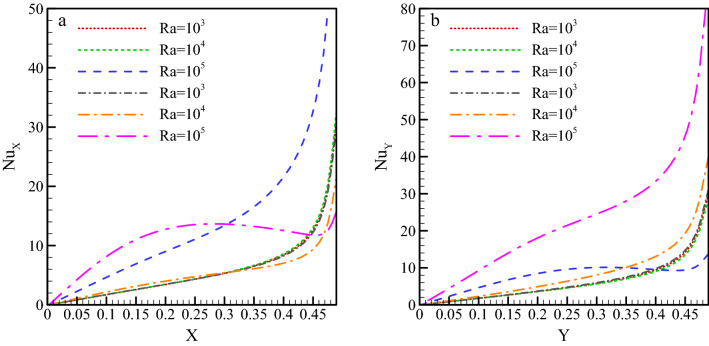
Variations in the local Nu along (**a**) the warm lower wall and (**b**) the warm left wall, for different Rayleigh numbers (dashed lines represent the 0° magnetic field angle and dash-dot lines represent the 90° magnetic field angle).

As shown in Fig. 5, Nu_ave_ is very low at the beginning of the walls and higher at the ends. This is due to the small diffusion of the fluid flow toward the corners of the cavity. The local Nu significantly rises with a rise in Ra number. This increase is more remarkable for regions of the wall with larger contact with vortices than the corners of the cavity. The highest Nu_ave_ on the lower wall occurs at a high Ra number and a magnetic field angle of 0°, and the highest Nu_ave_ on the right wall occurs at the same Rayleigh number and ω = 90°. The reason is that the magnetic field at these angles leads the fluid to move beside the wall without distancing from it, resulting in improved heat transfer.

**Figure 6:**
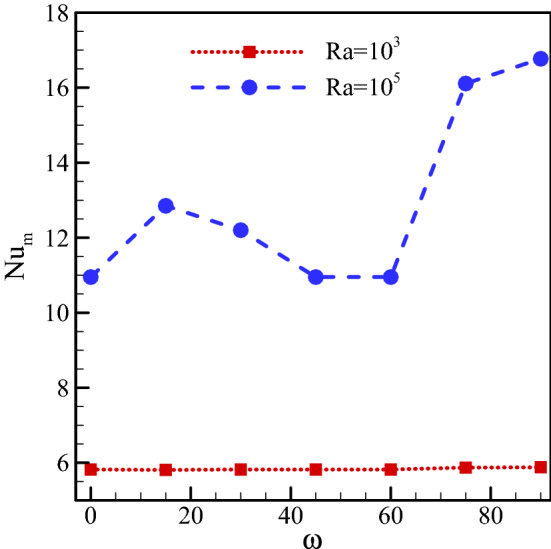
The variation of Nu_ave_ on the warm wall for $Ha=20, \, Rd=1, \, \varphi =0.03, \, \gamma ={45}^{\circ}$ and various Rayleigh numbers and magnetic field angles.

The Nu_ave_ on the warm wall has been drawn in Fig. 6 at $Ha=20, \, Rd=1, \, \varphi =0.03, \, \gamma ={45}^{\circ}$ and various Ra numbers and magnetic field angles. It is clear that Nu_ave_ has risen with rising Rayleigh number. As mentioned before, the buoyancy force, consequently, free convection increase with a rise in Rayleigh number. According to the temperature field, the increase in Rayleigh number has led to a higher concentration of isotherms near the differentially heated walls and higher curvature in these lines. This results in a larger temperature gradient and an improvement in free convection heat transfer. Heat transfer and Nusselt number increase with increasing temperature gradient. Variations in the magnetic field angle at low Ra numbers have not had a significant impact on Nu_ave_. However, high Rayleigh numbers have led to a rise and decrease in Nu_ave_ at various magnetic field angles. The change in ω affects the direction and intensity of vortex formation. At low Rayleigh numbers, the vortex formed in the cavity is weak and the fluid flow is not considerable. Changes in the magnetic field angle do not significantly influence this weak vortex. Consequently, thermal performance undergoes little change. Besides, ω has a considerable effect on the size and direction of the vortex at high Ra numbers. With a change in the velocity, the heat transfer through the walls changes. It is observed that the most suitable conditions for vortex formation and the highest Nu_ave_ exist at ω = 90°.

30 $${\text{Ra}} = 10^{3} \;\;\;\;\;{\text{Nu}}_{{\text{m}}} = { } - 2 \times 10^{ - 11} {\upomega }^{6} + 5 \times 10^{ - 9} {\upomega }^{5} - 4 \times 10^{ - 7} {\upomega }^{4} + 1 \times 10^{ - 5} {\upomega }^{3} - { }0.0001{\upomega }^{2} - 0.0007{\omega } + { }5.82$$

31 $${\text{Ra}} = 10^{4} \;\;\;\;\;{\text{Nu}}_{{\text{m}}} = { } - 4 \times 10^{ - 11} {\upomega }^{6} { } + 5 \times 10^{ - 7} {\upomega }^{5} { } - { }9 \times 10^{ - 6} {\upomega }^{4} { } + { }0.0004{\upomega }^{3} { } - { }0.0084{\upomega }^{2} { } + { }0.0596{\omega } + { }5.78$$

32 $${\text{Ra}} = 10^{5} \;\;\;\;\;\;{\text{Nu}}_{{\text{m}}} = { } - 2 \times 10^{ - 11} {\upomega }^{6} { } + 5 \times 10^{ - 7} {\upomega }^{5} { } - { }5 \times 10^{ - 7} {\upomega }^{ - 5} + { }0.0019{\upomega }^{3} { } - { }0.0443{\upomega }^{2} { } + { }0.4831{\omega } + { }10.95$$

**Figure 7:**
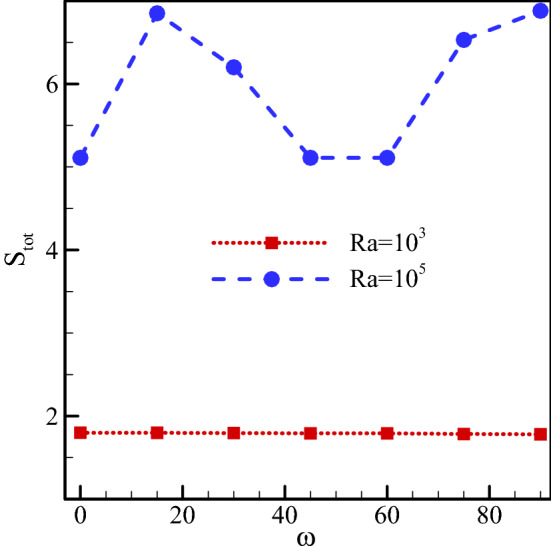
Total entropy generated for $Ha=20, \, Rd=1, \, \varphi =0.03, \, \gamma ={45}^{\circ}$ and various Ra numbers and magnetic field angles.

Total entropy generated for $Ha=20, \, Rd=1, \, \varphi =0.03, \, \gamma ={45}^{\circ}$ and various Ra numbers and ω are drawn in Fig. 7. The total generated entropy indicates the irreversibility in each state. The total generated entropy in this problem consists of three parts, namely the thermally generated entropy, the entropy generated via fluid loss, and the entropy generated by the magnetic field. The last term has the smallest contribution to the total entropy due to its small value compared to the other two entropies. This is due to the inverse relationship between the magnetic field and the velocity which causes a rise in the Ha number to decline the velocity. On the other hand, the thermal entropy and irreversibilities depend on the temperature and velocity gradients, respectively. The total generated entropy changes with variations in these two gradients. The change in the magnetic field angle does not influence the amount of entropy generated at low Ra numbers. The reason is that the temperature and velocity gradients do not change with a change in ω at low Rayleigh numbers. On the contrary, a change in ω at high Ra numbers results in a change in the velocity and temperature gradients, leading to a change in S_total_. In this state, the largest entropy generation occurs at ω = 90°. Moreover, there is a rise in the total generated entropy for cases of larger heat transfer due to the higher temperature gradient in these cases.

**Figure 8:**
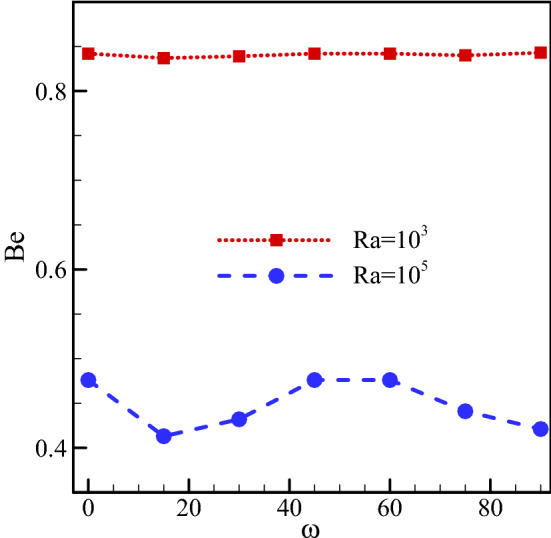
Bejan number for $Ha=20, \, Rd=1, \, \varphi =0.03, \, \gamma ={45}^{\circ}$ and various Rayleigh numbers and magnetic field angles.

The Bejan number for $Ha=20, \, Rd=1, \, \varphi =0.03, \, \gamma ={45}^{\circ}$ and various Ra numbers and ω are drawn in Fig. 8. The Bejan number is a criterion representing the thermal contribution of the S_total_. A larger Rayleigh number causes a rise in S_total_. This increase is due to a rise in both the viscous dissipation and the thermal contribution. As shown, the Bejan number falls as the Ra number rises. This is owing to the increase of the total generated entropy with rising Ra number. Since the generated entropy is in the denominator of the Bejan number fraction, its increase has led to a decrease in the Bejan number. Furthermore, a change in ω at low Rayleigh numbers does not impact the Bejan number because of its negligible effect on S_total_. However, due to the effect of changes in ω on the velocity and temperature gradients at high Rayleigh numbers, these changes can change the Bejan number. Variations in the total generated entropy have caused changes in the Bejan number, such that the lowest Bejan number has occurred at instances with the highest entropy generation.

### Variations in the radiation parameter and Ha number

**Figure 9:**
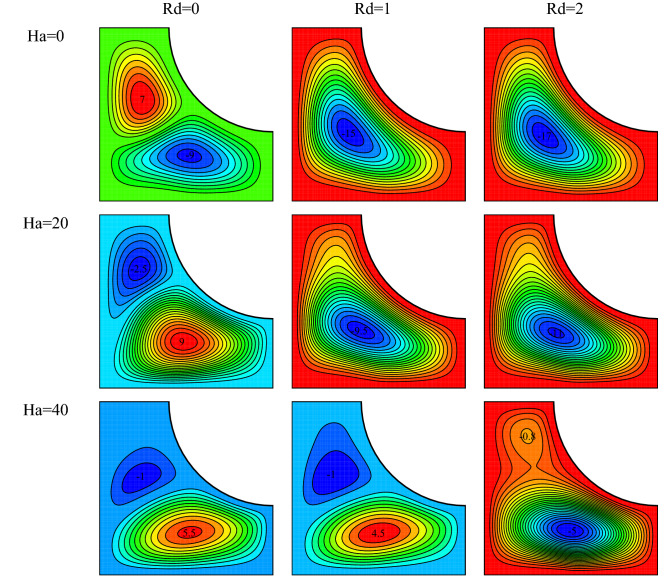
Flow field for $Ra={10}^{5}, \, \omega =0, \, \varphi =0.03, \, \gamma ={45}^{\circ}$ and various Hartmann numbers and radiation parameters.

Isotherms for $Ra={10}^{5}, \, \omega =0, \, \varphi =0.03, \, \gamma ={45}^{\circ}$ and various Hartmann numbers and radiation parameters are plotted in Fig. 9. The results show that the vortex velocity has reduced with increasing Hartmann number. A force denominated the Lorentz force somewhat prevents the formation of vortices is exerted on the cavity because of the magnetic field. Therefore, as shown, with a rise in the Ha number, the vortex tends more to the right side, diffusing more toward it, and reducing in size. Given the flow fields, a rise in Rd has resulted in a higher vortex velocity and has increased the stream function. The density difference increases with increasing heat transfer, and the rise in the buoyancy force causes the vortex to move faster. It is also observed that the direction of the vortex has changed at higher radiation parameters where heat transfer has increased. This is because the vortex tends to rotate clockwise at larger density differences where the buoyancy force is stronger.

**Figure 10:**
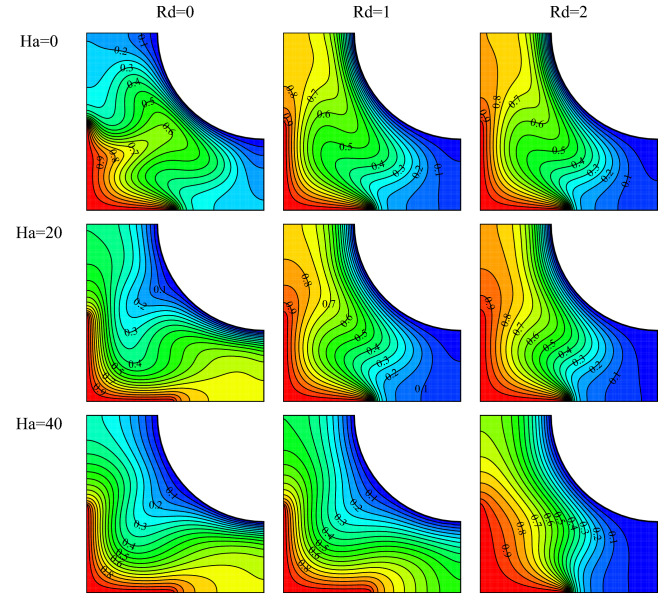
Temperature field for $Ra={10}^{5}, \, \omega =0, \, \varphi =0.03, \, \gamma ={45}^{\circ}$ and various Hartmann numbers and radiation parameters.

Isotherms for $Ra={10}^{5}, \, \omega =0, \, \varphi =0.03, \, \gamma ={45}^{\circ}$ and various Hartmann numbers and radiation parameters are drawn in Fig. 10. Larger Ha number causes a rise in the Lorentz force, which causes a reduction in the vortex velocity and, consequently, the weakening of convection. This phenomenon is witnessed by the decrease in the curvature of the isothermal lines at all radiation parameters. The cluttering in isotherms is declined by the increase in the Hartmann number indicating a rise in conduction. However, given the condition of the cavity in weak magnetic fields, the vortex does not diffuse considerably toward the right side of the cavity. With the strengthening of the temperature field and given the direction of the magnetic field, the vortex diffuses more rightward and has more contact with the cold wall. This can result in a rise in the Nu_ave_. A rise in the radiation parameter in the cavity leads to thermal performance improvement. This happens because of the addition of an extra source of heat transfer to the cavity in similar cases. However, for the present cavity and conditions, the rise in the radiation parameter changes the direction and the size of the vortex. This causes changes in the heat transfer conditions, affecting the temperature gradient and the curvature of isotherms under various conditions. In addition, the warming of the fluid due to a rise of the radiation parameter reduces the fluid diffusion toward the bottom of the cavity. As a result, warmer isothermal lines diffuse less toward the bottom of the cavity.

**Figure 11:**
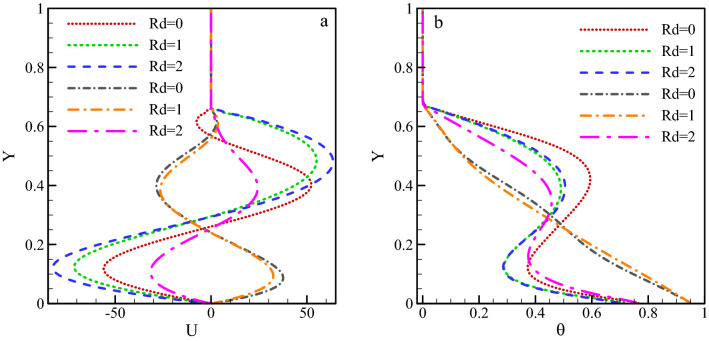
Variations of dimensionless (**a**) velocity and (**b**) temperature on line X = 0.5 for $Ra={10}^{5}, \, \omega =0, \, \varphi =0.03, \, \gamma ={45}^{\circ}$ and various radiation parameters.

The variation of dimensionless velocity and temperature on line X = 0.5 for $Ra={10}^{5}, \, \omega =0, \, \varphi =0.03, \, \gamma ={45}^{\circ}$ and various radiation parameters have been plotted in Fig. 11. Dashed lines correspond to the absence of the magnetic field, and dash-dot lines represent a strong magnetic field. The figure shows that, in the absence of a magnetic field, the dimensionless horizontal velocity has increased with increasing radiation parameter leads to the enhancement of Nu_ave_. With an increase in the density difference and, as a result, the buoyancy force, the vortex velocity rises. In a strong magnetic field, the vortex direction is seen to change with increasing radiation parameter. This causes a change in the direction of the dimensionless horizontal velocity. Also, the dimensionless temperature reduces with a rise in the radiation parameter in the absence of a magnetic field. This due to the decrease in the effect of vortices on the warm surface. In the presence of a magnetic field, the dimensionless temperature is seen to fall with rising radiation parameter due to the same reason. The velocity declines and the dimensionless temperature take almost linear form as a response to the magnetic field.

33 $${\text{Rd}} = 0\;\;\;\;\;y = 9 \times 10^{ - 6} Ha^{4} - 0.0008Ha^{3} + 0.0208Ha^{2} - 0.1631Ha + 7.59$$

34 $${\text{Rd}} = 1\;\;\;\;\;y = - 1 \times 10^{ - 5} Ha^{4} + 0.0012Ha^{3} - 0.0306Ha^{2} + 0.1266Ha + 13.38$$

35 $${\text{Rd}} = 2\;\;\;\;\;y = - 2 \times 10^{ - 17} Ha^{4} + 0.0002Ha^{3} - 0.0136Ha^{2} + 0.0052Ha + 17.63$$

**Figure 12:**
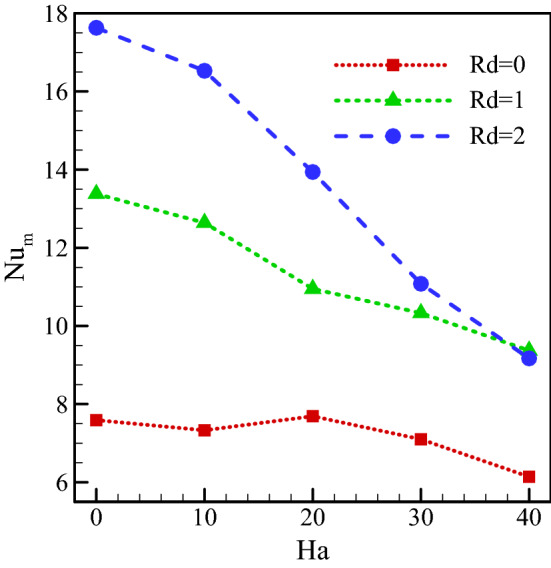
Average Nusselt number on the warm surface for $Ra={10}^{5}, \, \omega =0, \, \varphi =0.03, \, \gamma ={45}^{\circ}$ and various Hartmann numbers and radiation parameters.

The Nu_ave_ on the warm wall for $Ra={10}^{5}, \, \omega =0, \, \varphi =0.03, \, \gamma ={45}^{\circ}$ and various Hartmann numbers and radiation parameters are plotted in Fig. 12. A rise in Rd causes a higher heat transfer rate by introducing an additional heat transfer mechanism. In general, through the addition of radiative heat transfer, the fluid exchanges more heat, and the temperature difference in the cavity increases. This temperature difference leads to a larger buoyancy force and, consequently, a higher vortex velocity. In cases where conditions such as the addition of a magnetic field reduce the velocity inside the cavity, the fluid has a smaller diffusion toward the bottom. This leads to a smaller contact between the warm wall and the fluid, reducing the temperature gradient in that region. Hence, with a rise in Ha number in the presence of radiation, the Nu_ave_ decreases. A rise in the magnetic field in the absence of radiation results in two consequences. The first is a reduction in vortex velocity that results in lower Nu_ave_. This reduction in velocity is the result of the Lorentz force and a decline in the buoyancy force. The second is the larger diffusion of the fluid rightward. In the absence of a magnetic field, the vortex does not diffuse considerably toward the bottom of the cold wall. With a rise in Ha, the fluid diffuses more toward the right side. The heat transfer rate rises with rising contact between the fluid and the cold wall. As a result, a rise in the Hartmann number simultaneously increases and decreases heat transfer. This causes changes in Nu_ave_ versus the Hartmann number in the absence of radiation.

### Changes in the cavity angle and the nanofluid concentration

**Figure 13:**
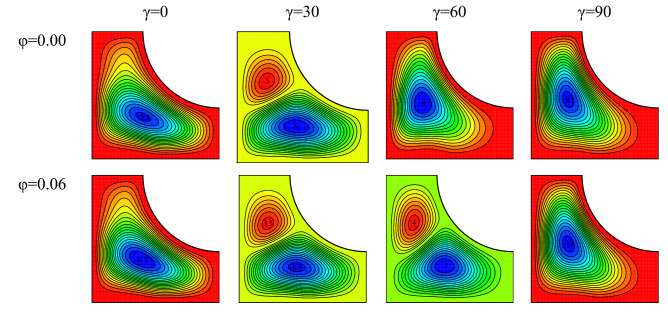
Flow field for $Ra={10}^{5}, \, \omega =0, \, Ha=20, \, Rd=1$ and various nanofluid concentrations and cavity angles.

Streamlines in various nanofluid concentration at $Ra={10}^{5}, \, \omega =0, \, Ha=20, \, Rd=1$ are plotted in Fig. 13. The nanofluid thermal conductivity rises with rising nanofluid concentration. Consequently, conductive and convective heat transfer rise. Therefore, the fluid near the constant-temperature walls reaches a temperature close to that of the walls, leading to a higher temperature difference and density difference leading to the improvement of the buoyancy force and the vortex velocity. The growth in the stream function value in the flow fields is a sign of this phenomenon. At certain cavity angles, a single vortex changes into two vortices with an increase in φ. The gravitational acceleration angle, the angle between the magnetic field and the cavity, and the conditions for the formation of vortices change with increasing cavity angle. It is therefore observed that the stream function value and even the number of vortices change with variations in the cavity angle.

**Figure 14:**
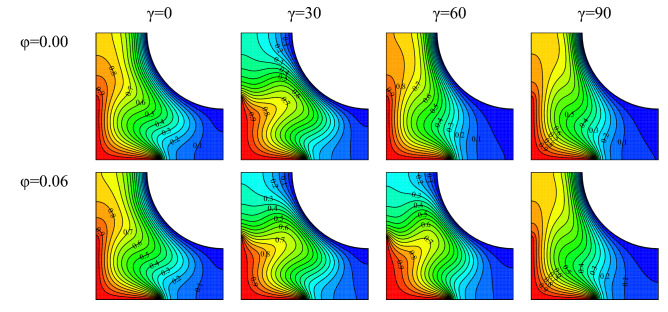
Temperature field for $Ra={10}^{5}, \, \omega =0, \, Ha=20, \, Rd=1$ and various nanofluid concentrations and cavity angles.

Isotherms for various nanofluid concentration at $Ra={10}^{5}, \, \omega =0, \, Ha=20, \, Rd=1$ are plotted in Fig. 14. With an increase in the nanofluid concentration, it is seen that the curvature of isotherms has slightly risen at some cavity angles. This has occurred due to the increase in vortex velocity and the enhancement of free convection in the cavity. Also, the curvature has risen at some angles and fallen at some other angles. The reason is the growth or reduction in vortex velocity with variations in the cavity angle. Furthermore, the temperature gradient has decreased near the warm wall. This results in a lower temperature gradient and Nu_ave_. The diffusion of the fluid toward the corners of the cavity reduces with the warming up of the fluid or a reduction in its velocity.

**Figure 15:**
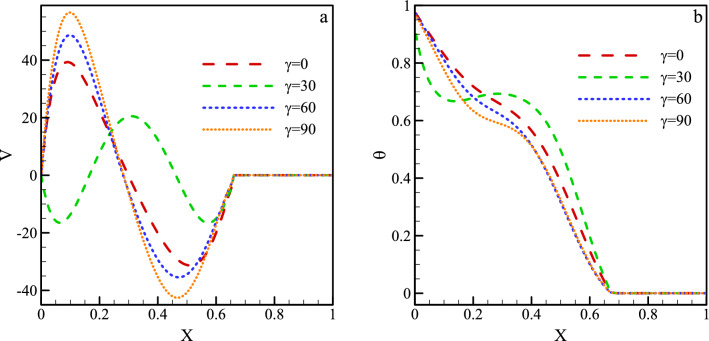
Variations of dimensionless (**a**) velocity and (**b**) temperature on line Y = 0.5 for $Ra={10}^{5}, \, \omega =0, \, Ha=20, \, Rd=1, \, \varphi =0.03$ and various cavity angles.

The variation of dimensionless velocity and temperature on line Y = 0.5 at $Ra={10}^{5}, \, \omega =0, \, Ha=20, \, Rd=1, \, \varphi =0.03$ and various cavity angles have been drawn in Fig. 15. As seen, the highest velocity corresponds to a vertical cavity. This state also corresponds to the strongest vortex. The conditions at this angle are the most suitable for vortex formation. These conditions are the positioning of warm and cold walls in various locations and the effect of $\omega$ on the flow pattern. Two vortices are formed in the cavity at a cavity angle of 30°. This is why two minimums are observed in the dimensionless vertical velocity values. Also, the temperature variations in this angle are more than in other cases due to the higher mix fluid flow in this cavity angle. The figure also shows that the temperature is almost linear at a cavity angle of 0°, and the curvature of the isothermal lines is highest at a cavity angle of 90°. At the section attached to the fin, the velocity is observed to tend to 0 with the dimensionless velocity also being 0.

**Figure 16:**
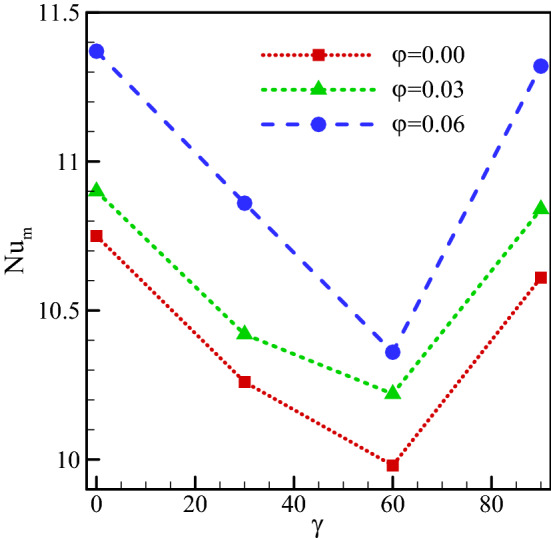
Average Nusselt number at the warm surface for $Ra={10}^{5}, \, \omega =0, \, Ha=20, \, Rd=1$ and various nanofluid concentrations and cavity angles.

The Nu_ave_ on the warm wall in various nanofluid concentrations and cavity angles at $Ra={10}^{5}, \, \omega =0, \, Ha=20, \, Rd=1$ are plotted in Fig. 16. As shown, Nu_ave_ in all cavity angles increases with increasing nanofluid concentration. Adding more nanopowder leads to higher thermal conductivity of the production. According to Eq. (25), the nanofluid thermal conductivity increases with a rise in the nanofluid concentration, resulting in a rise of the free heat convection according to the relationship of Nu_ave_. For all nanofluid concentrations, Nu_ave_ falls by rising the cavity angle up to 60°. By further rising the cavity angle, up to 90°, Nu_ave_ rises. The rise in the Nusselt number is dependent on the stream function value that represents the vortex velocity. Higher vortex velocities improve the free convection heat transfer.

36 $$\varphi = 0.0\;\;\;\;\;Nu_{m} = { }4 \times 10^{ - 6} \gamma^{3} { } - { }0.0003\gamma^{2} { } - { }0.0121\gamma { } + { }10.7$$

37 $$\varphi = 0.03{ }\;\;\;\;\;Nu_{m} = { }3 \times 10^{ - 6} \gamma^{3} { } - { }0.0001\gamma^{2} { } - { }0.0147\gamma { } + { }10.9$$

38 $$\varphi = 0.06\;\;\;\;\;Nu_{m} = { }9 \times 10^{ - 6} \gamma^{3} { } - { }0.0008\gamma^{2} { } - { }0.0011\gamma { } + { }11.37$$

**Figure 17:**
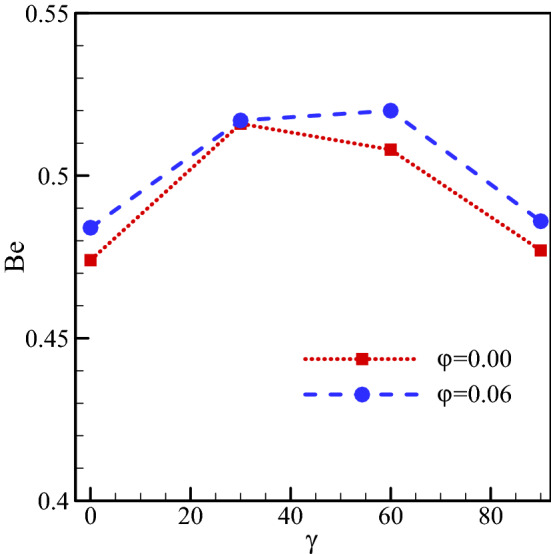
Bejan number for $Ra={10}^{5}, \, \omega =0, \, Ha=20, \, Rd=1$ and various nanofluid concentrations and cavity angles.

The Bejan number in various nanofluid concentrations and cavity angles at $Ra={10}^{5}, \, \omega =0, \, Ha=20, \, Rd=1$ are plotted in Fig. 17. As seen, the Bejan number rises with increasing nanofluid concentration. This increase occurs for all cavity angles, but it is larger for some angles and smaller for other angles. The temperature gradient rises with increasing nanofluid concentration, consequently, the thermal contribution of S_total_ increases leading to a rise in the Be number. As shown, for various cavity angles that the Bejan number is smaller when heat transfer is higher, and it is larger when heat transfer is lower. The stronger the vortex, the higher the heat transfer rate. In these cases, the velocity and temperature gradients are large, resulting in higher thermal and fluid loss entropies. Therefore, the Bejan number reduces with a rise in the total generated entropy.

## Conclusion

The MHD free heat convection in an AL_2_O_3_-water nanofluid-filled enclosure was investigated in this paper. Radiative heat transfer was included using the Roseland method, and the entropy generation was studied. The results are listed as:

1. Increasing the Rayleigh number increases the mean Nusselt number and the generated entropy and decreases the Bejan number.
2. The maximum heat transfer rate and entropy generation is obtained for the 90° magnetic field and the minimum value of the Bejan number for 15° magnetic field at high values of the Rayleigh number.
3. The variation of magnetic field angle has no significant effects on the heat transfer rate, generated entropy, and Bejan number at low values of the Rayleigh number.
4. In a weak magnetic field, the heat transfer rate increases by increasing the radiation parameter so that the Nusselt number can reach 10.04 in the absence of the magnetic field.
5. By increasing the Hartmann number at high values of the radiation parameter, the heat transfer is reduced in the cavity.
6. Adding nanoparticles to the base fluid leads to an increase in the mean Nusselt number. For example, the addition of 6% nanoparticles in the horizontal cavity increases the heat transfer rate by about 5.7%.
7. The maximum heat transfer rate occurs in the horizontal cavity and the minimum value in the cavity of 60° angle.

## Nomenclature

B_0_: Magnetic field strength
Be: Bejan number (–)
C_p_: Specific heat (J/kg K)
g: Gravitational acceleration (m/s^2^)
h: Convection heat transfer coefficient (W/m^2^ K)
H: Enclosure non-dimension length (–)
Ha: Hartmann number (–)
k: Thermal conductivity W/(m K)
l: Enclosure length (m)
L: Hot wall non-dimension length (–)
${\mathrm{Nu}}_{\mathrm{Y}}$: Local Nusselt number (–)
${\mathrm{Nu}}_{\mathrm{M}}$: Average Nusselt number (–)
$$\mathrm{P}$$: Pressure (Pa)
Pr: Prandtl number (–)
R: Cold fin non-dimension radius (–)
Rd: Radiation parameter (–)
Ra: Rayleigh number (–)
S: Entropy (W/K)
T: Temperature (K)
u, v: Velocity components in x and y direction (ms^−1^)
x, y: Cartesian coordinates (m)

## Greek symbols

$$\mathrm{\alpha }$$: Thermal diffusivity (m^2^ s^−1^)
${\varphi }$: Solid volume fraction (–)
$${\mathrm{k}}_{\mathrm{b}}$$: Boltzmann constant
$$\uptheta$$: Temperature (–)
$\upmu$: Dynamic viscosity (Pa s)
$$\mathrm{\vartheta }$$: Kinematic viscosity (m^2^ s^−1^)
$$\uprho$$: Density (kg m^−3^)
$$\upsigma$$: Electrical conductivity (Ω m)
$$\upgamma$$: Cavity angle (°)
*ω*: Magnetic field angle (°)

## Subscripts

c: Cold
f: Pure fluid
gen: Generation
h: Hot
nf: Nanofluid
s: Solid nanoparticles
tot: Total
